# The Involvement of the Chemokine RANTES in Regulating Luminal Acidification in Rat Epididymis

**DOI:** 10.3389/fimmu.2020.583274

**Published:** 2020-09-25

**Authors:** Xiao Feng, Bin-Fang Ma, Bo Liu, Peng Ding, Jin-Hua Wei, Pang Cheng, Sheng-Yu Li, Dong-Xu Chen, Zhi-Jian Sun, Zhen Li

**Affiliations:** ^1^Department of Human Anatomy, Histology and Embryology, Fourth Military Medical University, Xi’an, China; ^2^The General Hospital of Northern Theater Command, Shenyang, China

**Keywords:** RANTES, epididymis, epididymal epithelium, luminal acidification, macrophages, iNOS

## Abstract

**Background:**

A complex interplay between different cell types in the epithelium leads to activation of the luminal acidifying capacity of the epididymis, a process that is crucial for sperm maturation and storage. Basal cells sense the luminal angiotensin II (ANG II) and stimulate proton secretion in clear cells through nitric oxide (NO). Our previous study has shown the chemokine regulated upon activation normal T-cell expressed and secreted (RANTES) was expressed in the F4/80 positive macrophages of human epididymis. The objective of this study was to explore the involvement of RANTES in regulating the luminal acidification in the rat epididymis.

**Methods:**

The role of RANTES was investigated by *in vivo* perfusion with recombinant RANTES, Met-RANTES, and PBS of different pH values. Furthermore, rats vasectomy was performed to alter the epididymal luminal pH. RIA was used to measure the tissue homogenate ANG II concentration. Real time-PCR and western blot were employed to examine the expression levels of AGTR2, RANTES, CCR1, CCR5, and iNOS in epididymis.

**Results:**

RANTES was restricted to the basal macrophages of epididymal ducts and co-localized with its receptors CCR1 and CCR5. Both V-ATPase and iNOS were up-regulated in the cauda epididymis after perfused with recombinant RANTES, while the antagonist Met-RANTES perfusion led to a complete abrogation of the increased expression of V-ATPase in the apical membrane of clear cells and iNOS in macrophages. Upon alkaline perfusion, RANTES expression was significantly increased and the apical accumulation of V-ATPase in the clear cells was induced in the cauda epididymis. The luminal pH in the cauda epididymis increased after vasectomy. The concentration of the ANG II and the expression levels of AGTR2, RANTES, CCR1, CCR5, and iNOS dropped in the cauda epididymis following vasectomy.

**Conclusion:**

Upon the activation of basal cells, RANTES might induce the NO release from macrophages by interacting with its receptors, which increases proton secretion by adjacent clear cells. Thus, RANTES is possible to participate in the crosstalk among basal cells, macrophages and clear cells for the fine control of an optimum acidic luminal environment that is critical for male fertility.

## Introduction

The epididymis establishes a low luminal pH of 6.5–6.8 to maintain spermatozoa in a quiescent state during their maturation and storage in this organ (1). Acidification of the epididymal luminal fluid is critical for post-testicular maturation and acquirement of appropriate motility (2, 3), which is controlled by the intricate interaction of different types of cells in the highly specialized epididymal pseudostratified epithelium (4–6). Among the epithelial cells, angiotensin I (ANG I) is secreted by principal cells to the epididymal lumen (7), and is catalyzed by angiotensin-converting enzymes (ACE) to produce angiotensin II (ANG II) (8, 9). Then, ANG II binds with the receptor AGTR2, which is solely expressed on the surface of basal cells, stimulating the clear cells to increase the proton secretion via the activation of nitric oxide (NO)/cGMP pathway (4, 10). However, the intrinsic mechanism as to how AGTR2 activation regulates the NO production, still remains undiscovered.

Macrophages, as classical innate immune cells, play important roles in maintaining homeostasis such as phagocytizing foreign substances and secreting cytokines in the process of immunoreactivities (11, 12). In addition to their classical function in immune system, the non-classical function of macrophage in peripheral, non-lymphoid organs has been widely investigated (13). For example, in the small intestinal mucosal system (14, 15), macrophages can stretch out their long protuberances to the intestinal lumen to monitor the luminal composition changes so as to regulate the absorption of nutrients and secretion of goblet cells by interacting with intestinal epithelial cells (16, 17). Similar to the intestinal tract, macrophages in the initial segment of epididymis also extend long slender projections to the epididymal lumen to sample the fluid components in the lumen (18, 19). Besides, there still exists another type of macrophages whose morphology is distinct from those with long projections, presenting basal round-like distribution in the distal segments of the epididymis (4). The function of this subtype of macrophages still remain unknown.

The chemokine regulated upon activation normal T-cell expressed and secreted (RANTES) was initially recognized as a kind of chemo-attractant for immune cells (20–23). The receptors of RANTES include high affinity receptors CCR1 and CCR5, and low affinity receptor CCR3, all of which belong to seven transmembrane G protein coupled receptor family (22, 24). With the researches deepening, RANTES have been found to possess some new biological characteristics (25), such as facilitating breast tumor progression and metastasis (26, 27), and triggering drug-resistance in breast, ovarian, and prostate cancers (28–30). Our previous work has demonstrated that RANTES was mainly expressed in F4/80 positive macrophages in the basal region of human epididymal epithelium (31). RANTES has also been proved to promote the activity of macrophages by increasing their production of NO (32). Furthermore, it has been reported that the expression of RANTES can be promoted by ANG II in aorta (33) and glomerular endothelial cells (34).

Based on the aforementioned evidence, we hypothesized that RANTES expressed in the epididymal basal macrophages might be involved in the crosstalk between basal cells and clear cells when regulating the luminal acidification. To address this hypothesis, we examined the expression of RANTES and its receptors in rat epididymis and further investigated whether RANTES function in the acidification of the luminal fluid by *in vivo* perfusion and vasectomy model.

## Materials and Methods

### Animals

Adult male Sprague Dawley (SD) rats were purchased from the Laboratory Animal Center of the Fourth Military Medical University. The animals were housed in individual cages and fed with food and water *ad libitum*. They were kept in a controlled environment on a 12 h/12 h light/dark cycle. The adult rats (12-week-old) were procured. Experimental procedures were approved by the local ethical committee.

### Vasectomy

Adult male rats were distributed randomly into two groups: 8 weeks sham vasectomy (*n* = 7) and 8 weeks bilateral vasectomy (*n* = 7). Surgical procedures of animals vasectomy was accomplished as reported previously (35). The operative process was completed to minimize postoperative adhesion and inflammation. The surgery was performed under sodium pentobarbital anesthesia [60 mg/kg body weight, intraperitoneally (i.p.)].

### *In vivo* Perfusion of the Distal Cauda Epididymis

Adult male Sprague-Dawley rats were anesthetized with sodium pentobarbital as described above. The vas deferens was cannulated through the lumen with a micro cannula (0.31 mm OD, 0.16 mm ID; Anilab Software & Instruments Co., PE-0402) connected to a 1-ml syringe. A small incision was made in the distal cauda epididymal region to allow the perfusate to exit the tubule. Perfusion was performed retrogradely at a rate of 20 μl/min using a syringe pump (78-0120S, Stoelting). The lumen was initially washed free of sperm with PBS (0.01M, pH 6.8) for usually 10 min. Then, vas deferens and cauda epididymis were perfused with recombinant RANTES (PeproTech, United States), Met-RANTES (R&D Systems, United States), and PBS of different pH values, respectively. The perfusion was performed retrogradely at a rate of 5 μl/min, and the luminal solution was sustained for 1 h. At the end of the experimental period, the cauda epididymis was harvested and extracted for the RNA and the protein. Or tissues were then washed in PBS, pH 7.4, and perfused with a solution containing 4% paraformaldehyde for 15 min for frozen sections.

### Semi-Quantitative RT-PCR and Real-Time Quantitative RT-PCR

Total RNA was extracted from the caput, corpus, and cauda regions of the rat epididymis, using TRIzol Reagent (Takara, Shiga, Japan) according to the supplier instructions, and semi-quantitative RT-PCR for RANTES, CCR1, CCR3 and CCR5 were performed as described previously (36). Real-time quantitative PCR analysis of V-ATPase, RANTES, CCR1 and CCR5, iNOS, AGTR2 expression in the different epididymal regions were performed with the MiniOpticon system (Bio-Rad, Hercules, CA, United States). Each reaction was performed in triplicate by using 10 ng of cDNA from each sample and SYBR Primix Ex Taq (TaKaRa, Shiga, Japan). The relative abundance of target transcript was quantified using the comparative Ct method. The **β** -actin or GAPDH from the same exacts were used as internal control. Data obtained from three independent experiments was calculated. Primers for PCR and qPCR were designed according to the rat sequences found in GenBank (Supplementary Tables 1, 2).

### Western Blotting

The protocol for protein extracts from rat epididymis was designed, based on previously described procedures (36). Sample aliquots containing equal amounts of protein were separated via SDS-PAGE and transferred onto polyvinylidene difluoride membranes. The membranes were blocked in 5% non-fat milk for 1 h at room temperature, and incubated overnight at 4°C with rabbit anti-RANTES (Abcam, Cambridge, United Kingdom), rabbit anti-V-ATPase (GeneTex, Irvine, CA, United States), rabbit anti-iNOS (Abcam, Cambridge, United Kingdom), rabbit anti-AGTR2 (GeneTex, Irvine, CA, United States), rabbit anti-CCR1 (Abcam, Cambridge, United Kingdom), rabbit anti-CCR5 (GeneTex, Irvine, CA, United States), mouse anti-**β**-actin (Invitrogen, United States) and mouse anti-GAPDH (Invitrogen, United States). The membranes were then washed with TBST buffer and incubated with an appropriate secondary antibody (HRP-conjugated anti-rabbit IgG or HRP-conjugated anti-mouse IgG, Sigma, St. Louis, MO, United States) for 2 h at room temperature. After extensive washing, the densities of labeled protein bands on the blots were detected using an enhanced chemiluminescence reagent (Thermo, Rockford, IL, United States) and captured using a ChemiDoc MP System (Bio-Rad, Hercules, CA, United States).

### Immunohistochemistry

The avidin-biotin technique was applied as previously described (31). After H_2_O_2_ treatment, paraffin-embedded sections were blocked for 30 min with 10% donkey serum at room temperature and incubated with the related anti-RANTES primary antibodies (5 mg/mL) overnight at 4°C, then followed by the 1:300-diluted biotinylated secondary antibody. The negative controls were performed in the absence of primary antibody. The same method was used in the experiment to detect the localization of CCR1 and CCR5 in rat epididymis, where the goat anti-CCR1 antibody and goat anti-CCR5 antibody (Santa Cruz Biotechnology) were used, with secondary antibodies, biotinylated anti-goat IgG (Sigma, St. Louis, MO, United States), applied for immunohistochemical staining.

### Tissue Fixation and Immunofluorescence

Fixed tissues were dehydrated in 30% sucrose for 2 h at room temperature, embedded in Tissue-Tek OCT compound, and mounted and frozen on a cutting block. Tissues were cut in a Reichert Frigocut microtome at 10 μm thickness and sections were placed onto Superfrost Plus microscope slides. Sections were rehydrated in PBS for 10 min and pretreated with 1% (w/v) SDS. After three washes in PBS, slides were preincubated in 1% (w/v) BSA in PBS with 0.02% Na-azide for 30 min to block non-specific staining, after which they were incubated with different primary antibodies for 2 h at room temperature. The antibody included mouse anti-V-ATPase (Santa Cruz Biotechnology, Dallas, TX, United States) and rabbit anti-iNOS (Abcam, Cambridge, United Kingdom). Subsequently, the sections were incubated with an appropriate secondary antibody (FITC-conjugated goat anti-mouse, or Cy3-conjugated goat anti-rabbit, Beyotime, Shanghai, China) for 2 h at room temperature in the dark. Double Immunofluorescence Labeling with rabbit anti- RANTES antibody and rat anti-F4/80 antibody (Abcam, Cambridge, United Kingdom), the rabbit anti-RANTES antibody and goat anti-CCR1/CCR5 antibody, the goat anti-iNOS antibody and rat anti- F4/80 antibody, followed by the mixed secondary antibodies (Alexa 488-conjugated goat anti-rabbit, or Cy3-conjugated mouse anti-goat, Invitrogen, United States). The sections were then mounted in 50% glycerol and examined with the fluorescence microscope (Zeiss, Jena, Germany).

### Statistical Analysis

All the results were expressed as mean ± SEM of independent experiments repeated for at least three times. Statistical analysis was performed with GraphPad Prism 5 software. Relative mRNA levels in different epididymal regions were compared by one-way ANOVA followed by Dunnett’s multiple comparison test. Comparisons between two groups were carried out by Student’s *t*-test. *P*-values < 0.05 were accepted as significant.

## Results

### The Expression Profile of RANTES and Its Receptors in the Rat Epididymis

**FIGURE 1:**
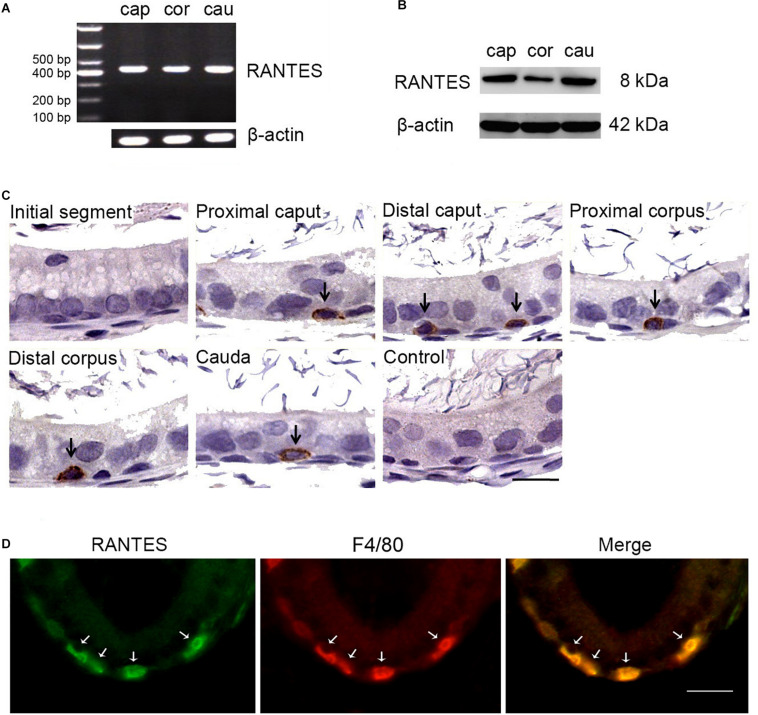
Expression and cellular localization of RANTES in the adult rat epididymis. **(A)** RT-PCR analysis of RANTES in the caput (Cap), corpus (Cor) and cauda (Cau) regions of the epididymis. The PCR products were found in all three regions. **(B)** Protein extracted from different segments of the epididymis was analysis by WB. RANTES was detectable in the caput (Cap), corpus (Cor) and cauda (Cau). **(C)** Immunohistochemistry of RANTES in rat epididymis. Positive reactions were found restricted in partial basolateral cells (black arrows) of the epididymal ducts. **(D)** The cellular location of RANTES in cauda epididymis was detected by double IF. The green signal of RANTES (Alexa 488 labeled) coincide with the red signal of F4/80 (Cy3 labeled). White arrows: positive signals for RANTES or F4/80. Bar = 20 μm.

As a first step to determine the regional expression profile of RANTES in the rat epididymis, RT-PCR was performed in different segments and 430 bp positive products, the result of which indicated that RANTES was ubiquitously expressed in caput, corpus and cauda segments (Figure 1A). Moreover, the results of Western Blot analysis using the specific antibody against RANTES also confirmed the wide expression of RANTES in different segments of rat epididymis (Figure 1B). The immunohistochemical staining was carried out to elucidate the accurate localization of RANTES in the epithelium. Consistent with previous results (31), RANTES was found mainly distributed in the basal compartment of the caput, corpus and cauda segments while no detectable positive signals was found in the initial segment and in negative control (Figure 1C). Considering the specific morphology of these positive cells, which appear to be hemispherical, adhere to the basement membrane and locate underneath the columnar epithelial cells, we postulate that these cells could either be basal cells or basal macrophages. To further confirm the cell types that express RANTES, macrophage markers, F4/80 antibody, was stained by immunofluorescence to distinguish the two cell types, showing the location of RANTES particularly in the macrophages (Figure 1D).

**FIGURE 2:**
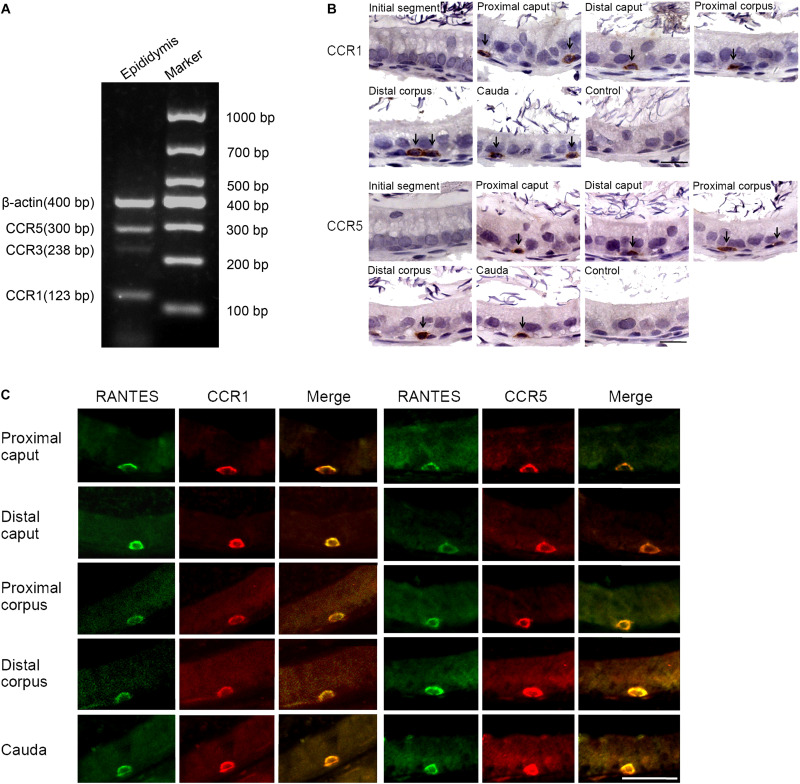
Expression of RANTES receptors CCR1, CCR3 and CCR5 in the adult rat epididymis. **(A)** Expression of CCR1, CCR3 and CCR5 in rat epididymis were detected by RT -PCR. **(B)** Immunohistochemistry of CCR1 and CCR5 in rat epididymis. Black arrows: positive signals for CCR1 or CCR5. **(C)** Double IF staining was applied to detected the co-localization of RANTES with CCR1 and CCR5 in rat epididymis. The green signal of RANTES (Alexa 488 labeled) coincide with that of the red signal of CCR1 and CCR5 (Cy3 labeled). Bars = 20 μm.

Several membrane receptors such as CCR1, CCR3, and CCR5 have been validated to be responsible for transducing the signal of RANTES in a context-dependent manner in different cellular processes (29). To identify the specific downstream receptors of RANTES in rat epididymis, we detected the expression profile of CCR1, CCR3 and CCR5 in the epididymis. We found the remarkable expression of CCR1 and CCR5 and the weak expression of the CCR3 in rat epididymis (Figure 2A). Similar to the distribution pattern of RANTES, the expression of CCR1 and CCR5 were mainly observed as positive brown granules in the basal compartment of the caput, corpus and cauda regions but disappeared in the initial segment and negative control (Figure 2B). The confocal imaging results also showed that both CCR1 and CCR5 proteins were co-localized with RANTES in the macrophages along the epididymis duct (Figure 2C). Thus, in rat epididymis, RANTES and its receptors CCR1 and CCR5 are predominantly expressed in the F4/80 positive macrophages which are located at the basal compartment of caput, corpus and cauda segments rather than the macrophages localized at the initial segment.

A significant difference was found in the morphology between the macrophages localized at the initial segment and those in other segments of the epididymal epithelium. The macrophages with long processes in the initial segment were mainly distributed in the adluminal compartment, while those in other segments of the epididymis exhibited basal round-like cells. No significant functional difference between these two subsets of macrophages was reported in previous studies. The unique localization of RANTES and its receptors in basal macrophages may indicate the difference of RANTES-mediated function between the two subtypes of macrophages which will be discussed later in the section “Discussion.”

### RANTES Induces the Epididymal Luminal Proton Secretion

**FIGURE 3:**
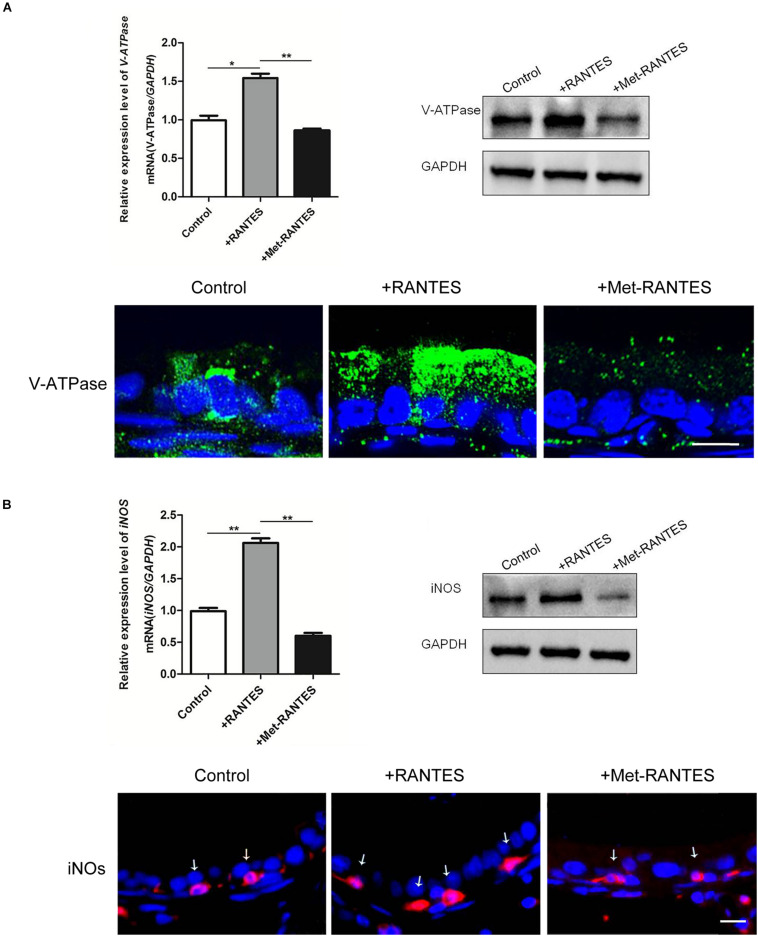
Effects of RANTES and Met-RANTES on V-ATPase and iNOS in perfusion model. Rat cauda epididymis were perfused luminally *in vivo* with recombinant RANTES and Met-RANTES. Real-time PCR and WB analysis of the mRNA and protein levels of V-ATPase **(A)** and iNOS **(B)** were up-regulated by the addition of RANTES compared to the control group (pH of 6.8), but decreased with the antagonist Met-RANTES replenishment. Immunofluorescence staining showed a notable accumulation of V-ATPase in the apical membrane of clear cells **(A)** and a strong signal of iNOS (white arrows) in the epididymis **(B)** by the addition of RANTES, whereas it decreased with Met-RANTES replenishment. **P < 0.01, *P < 0.05, *N* = 5–6, Bar = 20 μm.

In the epididymis, epithelial cells work in a concerted manner to establish a luminal acidic milieu which is essential for the post-testicular maturation and storage of spermatozoa. To examine whether RANTES is involved in the regulation of the unique acidic microenvironment in epididymal lumen, *in vivo* cauda microperfusion model was used to further determine the direct effects of RANTES on the luminal acid-base state. In the epididymal epithelium, V-ATPases in the clear cells are the final effectors which are responsible for the proton secretion and are sensitive indicators to evaluate the state of acid secretion. The effects of recombinant RANTES and Met-RANTES on V-ATPase were then examined in the *in vivo* perfusion model. After RANTES perfusion, the expression of V-ATPase was significantly up-regulated compared to the control group (pH of 6.8). Meanwhile, the immunofluorescent staining showed a notable accumulation of V-ATPases in the apical membrane of clear cells. Yet, the expression levels of V-ATPase were decreased with Met-RANTES replenishment compared with those in the RANTES perfusion group. Correspondingly, the immunofluorescent staining indicated that the accumulation of V-ATPases in the apical membrane of clear cell disappeared (Figure 3A).

Previous studies have elaborated that the expression of proton pumping V-ATPase in the apical membranes of epididymal clear cells is remarkably up-regulated via the activation of NO/cGMP pathway, leading to increased acid secretion. INOS is the enzyme which is responsible for NO production and used to assess the cellular ability to produce NO (37). We found that iNOS was also expressed in the F4/80 positive macrophages in the epididymal lumen (Supplementary Figure 1). So, it is possible that the intrinsic mechanism of RANTES regulates the expression of V-ATPases through the activation of NO/cGMP pathway. Real-time PCR and Western blot demonstrated that both mRNA and protein of iNOS were up-regulated by the addition of RANTES compared to the control group (pH of 6.8). Meanwhile, the immunofluorescent staining showed a stronger signal of iNOS in the macrophages. And the expression level of iNOS decreased with Met-RANTES replenishment compared with those in the RANTES perfusion group. The immunofluorescent staining, correspondingly, indicated weakened signal intensity of iNOS in macrophages (Figure 3B).

### The Aberrant Alkaline Luminal Environment Induces the Expression of RANTES and Proton Secretion

**FIGURE 4:**
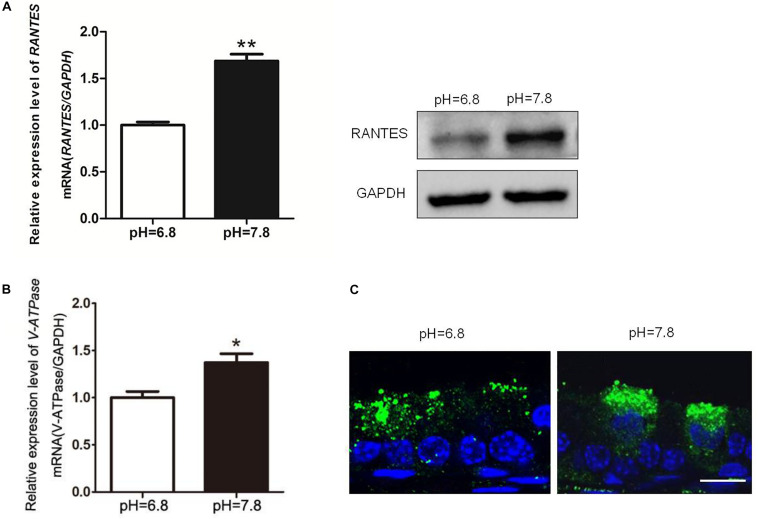
Changes of RANTES and V-ATPase upon rat cauda epididymis alkaline perfusion. Rat cauda epididymal lumen was perfused *in vivo* with PBS (pH values 6.8 or 7.8). **(A)** Real-time PCR and WB revealed that upon alkaline treatment (pH of 7.8), the expression level of RANTES in cauda epididymis was significantly up-regulated compared with that in the control group (pH of 6.8). Meanwhile, **(B)** expression of V-ATPase mRNA was increased examined by real-time PCR, *N* = 5–6. **(C)** The Results of CLSM showed that the alkaline pH also induced the apical accumulation of V-ATPase in the clear cell. **P < 0.01, *P < 0.05, *N* = 5–6, Bar = 20 μm.

While the pH values of the epididymal lumen in different segments are maintained at a relative stable state, the intrinsic mechanism still remains uncertain. To examine RANTES’ role in maintaining the acidic environment, we used the alkaline perfusion model (PBS at pH 7.8) to simulate the luminal acid-base fluctuation. Compared with the control group (pH 6.8 PBS), the expression of RANTES in cauda epididymis were remarkably enhanced after perfusion with pH 7.8 PBS (Figure 4A). Besides, we also observed significantly upregulated mRNA of V-ATPase in cauda epididymis (Figure 4B), and intensive enrichment of V-ATPase in the apical membranes of clear cells under the alkaline condition, suggesting the induction of luminal acid secretion after alkaline microperfusion (Figure 4C). Therefore, we confirmed that abnormal alkaline environment could induce the production of RANTES, which could in turn promote acid secretion to restore the original acidic microenvironment.

### RANTES Is Involved in the Abnormal Luminal Acid-Base State in the Vasectomy Model

**FIGURE 5:**
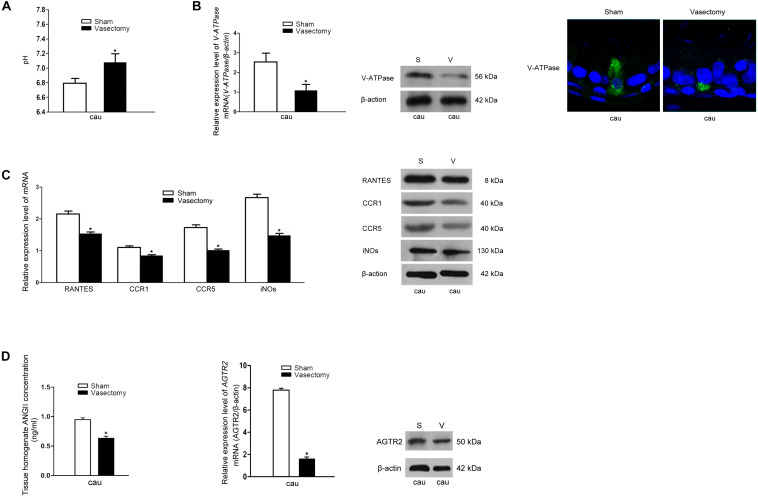
Effects of rat vasectomy on the luminal pH and the expression of V-ATPase, RANTES, CCR1, CCR5, iNOS, and RAS. **(A)** 8 weeks after rat bilateral vasectomy, luminal fluid pH of cauda epididymis increased and remained alkaline. **(B)** The expression of V-ATPase in rat cauda epididymis was detected by Real time-PCR, WB and IF staining. **(C)** 8 weeks after rat bilateral vasectomy, the expression levels of RANTES, CCR1, CCR5, and iNOS mRNA and protein in cauda epididymis were decreased revealed by Real time-PCR and WB. **(D)** The tissue homogenate ANG II concentration were measured by RIA and the expression of AGTR2 was detected by Real time -PCR and WB. *P < 0.05, *N* = 5–6, Bar = 20 μm.

Vasectomy is a classic surgical intervention for male contraception, which is also one of the frequently used animal models for the research into male reproductive function. Previous research has reported remarkable increase of lumen pH after vasectomy the yet no report regarding the underlying mechanism was found (35). Therefore, we constructed a rat vasectomy model to investigate whether RANTES is involved in the abnormal acid-base changes after vasectomy. Interestingly, after the vasectomy, we did find some prominent changes in the epididymal lumen. First, consistent with previous studies, the pH value of cauda in the experimental group was significantly higher than that in the sham-operated group (Figure 5A). Besides, the expression of V-ATPases in the apical membranes of clear cells was found decreased in the vasectomy group compared with the sham-operated group (Figure 5B), and iNOS was also found to decline in the cauda (Figure 5C). Finally, by real-time PCR and Western blot analysis were performed to detect the expression levels of RANTES and its receptors and we found both of them decreased significantly in the cauda segments (Figure 5C), suggesting the down-regulated expression of RANTES as a potential factor causing decreased acid secretion.

Though our research confirmed that aberrant RANTES expression could account for the abnormal outcome of alkaline luminal fluid after vasectomy, no investigation was done to reveal the upstream mechanism regulating RANTES expression. Recent reports showed that ANG II could stimulate V-ATPase-dependent proton extrusion and NO could be produced in the macrophages and function as one of the downstream effectors of AGTR2. These clues suggest that RANTES could be responsible for the transduction of ANG II/AGTR2 signal pathway in regulating NO synthesis. To further investigate the upstream pathway of RANTES, we measured the concentration of ANG II and the expression of AGTR2 in the vasectomy group and control group. As expected, the results showed a clear-cut demotion of ANG II in the vasectomy group (Figure 5D). Real-time PCR and Western blot were performed to detect the expression of AGTR2 and the data presented declined mRNA and protein of ANG II in the cauda of epididymis (Figure 5D). The results indicated that the decreased expression of RANTES may attribute to the abrogation of ANG II/AGTR2 signals but the direct regulatory relation between them needs to be validated in further experiments.

## Discussion

The results from the present study showed that RANTES and CCR1, CCR5 were constitutively expressed in the basal macrophages of rat epididymis, and played an essential role in the process of acidification in the luminal milieu. RANTES up-regulated the expression of iNOS in the macrophages and V-ATPase in the apical membrane of clear cells. Moreover, the expression of RANTES decreased and the pH value of the epididymal lumen increased following vasectomy.

In the epididymis, macrophages are widely distributed in different segments, exhibiting unique phenotypes and function to establish and maintain an appropriate environment for sperm maturation and storage (18, 38, 39). Epididymal macrophages can be divided into two subsets according to their distinguished appearances. Intraepithelial macrophages in the initial segment extend slender projections through the epithelial cells toward the lumen. This unique morphological characteristic was not observed in macrophages from the basal compartment of distal regions. Macrophages in the initial segment is mainly responsible for presenting antigen to the lymphocytes and eliminating defective epithelial cells and abnormal spermatozoa in epididymis, as well as the defense reaction in luminal infection (19, 40–42). Yet, the function of macrophages restricted to the basal region of the tubule is still unclear. Our study found that RANTES and its receptors CCR1 and CCR5 were specifically expressed in these basolateral macrophages, while they were absent in the initial segment. Thus, RANTES-positive macrophages might display some functions distinctive from those with long projections in the initial segment through its receptors CCR1 and CCR5.

In addition to the well-known chemotactic function, the non-canonical function of RANTES in sperm maturation seems more complicated than we previously thought (41). The present study showed the direct effects of RANTES on regulating the luminal acidification in the epididymis. RANTES perfusion induced the accumulation of V-ATPase in the apical membrane of clear cells and the expression of iNOS in the macrophages. A potent RANTES receptor antagonist Met-RANTES (43) was used to address the role of RANTES signaling through its receptors CCR1 and CCR5. Met-RANTES perfusion led to a complete abrogation of the increased expression of V-ATPase and iNOS in the epididymal epithelium. Since RANTES is a small protein of 8-kDa, we postulate that luminal perfused RANTES might directly bind to its specific 7-transmembrane G protein-coupled receptors, namely CCR1 and CCR5, on the surface of basal macrophages in the epithelium. Additional experiments such as using the methods of confocal microscopy and western blot should be done in the follow-up studies. It has been shown that proton was secreted by epididymal clear cells via activation of the NO/cGMP pathway (10). Therefore, RANTES might stimulate proton secretion by epididymal clear cells via activation of the NO released from macrophages. On the other hand, in the experiments detailed here, alkaline PBS induced the expression of RANTES within the epididymis and the accumulation of V-ATPase in the apical membrane of clear cells, indicating that the expression of RANTES could be increased responsively upon the exogenous luminal alkaline treatment in the epididymis.

We utilized the vasectomy model to further explore the function of RANTES in the epididymis. The luminal pH in the cauda epididymis increased as compared to control by 8 weeks post vasectomy, consistent with previous reports that luminal pH in the proximal cauda epididymis was significantly more alkaline (35). Accordingly, the accumulation of V-ATPase in the apical membrane of clear cells was decreased. Interestingly, the expression levels of RANTES, CCR1, CCR5, and iNOS were all significantly reduced in the cauda epididymis following vasectomy. This observation suggests that the decreased expression of RANTES and its receptors in the cauda epididymis may be one of the causes of the luminal alkaline pH in the vasectomy model. Previous study revealed that vasectomy altered the renin-angiotensin system (RAS) expression in the epididymis (7), which is coincided with our findings that both ANG II and AGTR2 decreased significantly in the cauda epididymis following vasectomy. It has been reported that ANG II promoted the expression of RANTES in glomerular endothelial cells through AGTR2 (34), and that AGTR2 was solely expressed in basal cells in the epididymal epithelium (10). All these studies strongly point to ANG II/AGTR2 as a factor regulating RANTES expression in the cauda epididymis to maintain the acidity of luminal fluid.

## Conclusion

**FIGURE 6:**
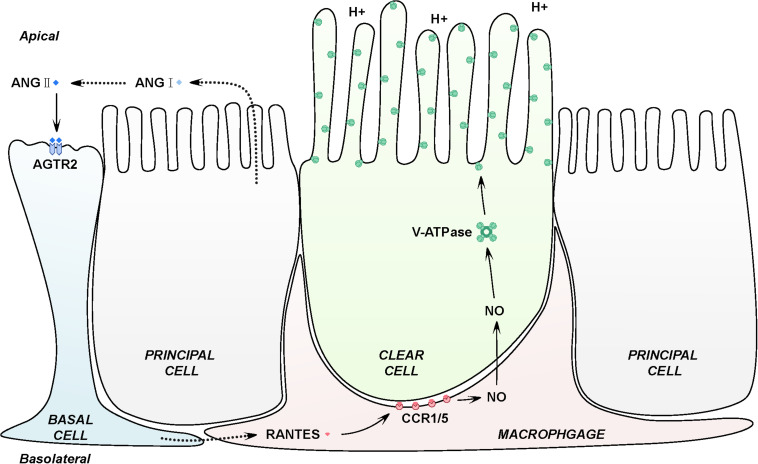
Schematic representation of RANTES in the basal macrophages contributing to the intricate luminal acidic regulation in the epididymis. Firstly, epididymal luminal ANG II binds with its receptors AGTR2 expressed on the membrane of basal cells, inducing the expression of RANTES and its receptors, CCR1 and CCR5 in the basal macrophages. Secondly, RANTES could function with CCR1 and CCR5 to increase the expression of iNOs, which will lead to the up-regulation of NO. Finally, NO will diffuse to the adjacent clear cells to stimulate the expression of V-ATPase, causing the increased secretion of proton to the epididymal lumen to maintain the normal acidic microenvironment.

In conclusion, our findings indicate that macrophages at the basal part of epididymal duct facilitates the luminal acidification via RANTES. In another words, activation of AGTR2 in basal cells by luminal ANG II may stimulate RANTES in basal macrophages to induce the production of NO, which results in the accumulation of V-ATPase and increases proton secretion by adjacent clear cells (Figure 6). This study highlights a novel biological activity for RANTES, a chemokine best known for the ability to induce directional cellular recruitment. By participating in the crosstalk among basal cells, macrophages and clear cells, RANTES may regulate acidic luminal environment that is critical for male fertility.

## Data Availability Statement

All datasets presented in this study are included in the article/Supplementary Material.

## Ethics Statement

The animal study was reviewed and approved by the Research Ethics Committee of the Fourth Military Medical University.

## Author Contributions

ZL, XF, and B-FM: conceived and designed the experiments. XF, B-FM, J-HW, PC, S-YL, and D-XC: performed the experiments. ZL, XF, B-FM, BL, and PD: analyzed the data. ZL and Z-JS: contributed to reagents, materials, and analysis tools. ZL, XF, and BL: wrote the manuscript. All authors contributed to the article and approved the submitted version.

## Conflict of Interest

The authors declare that the research was conducted in the absence of any commercial or financial relationships that could be construed as a potential conflict of interest.
