# Supplementary figures and images for: The Involvement of the Chemokine RANTES in Regulating Luminal Acidification in Rat Epididymis
Source: Front Immunol. 2020 Sep 25;11:583274. doi: 10.3389/fimmu.2020.583274 (PMC7544837; doi:10.3389/fimmu.2020.583274)

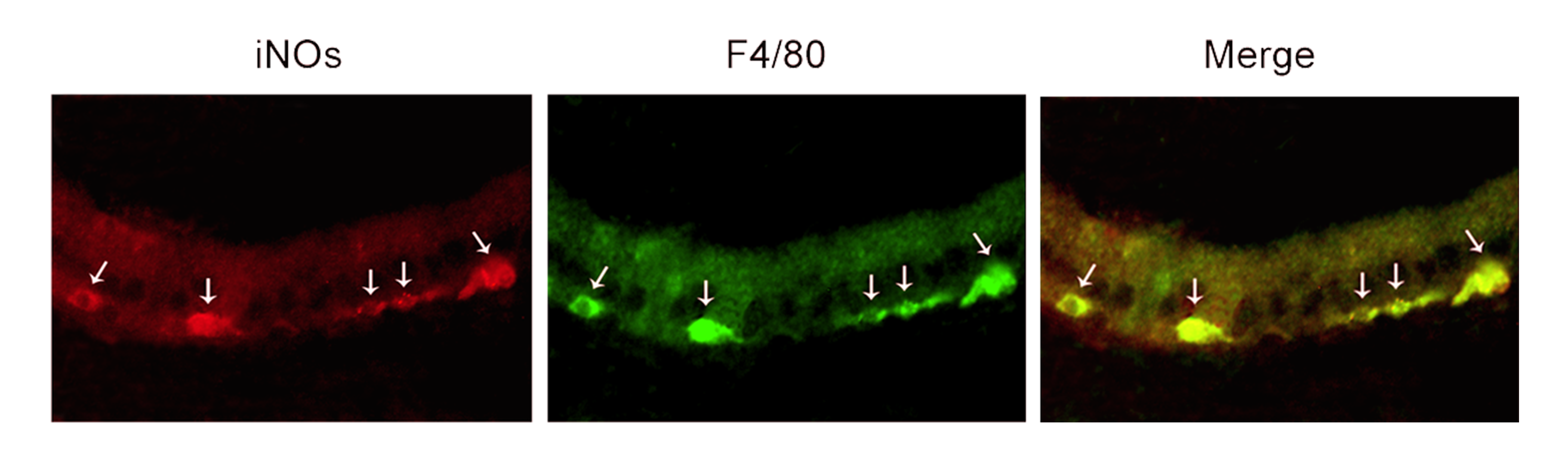

Supplement: Supplementary file 1 [file Image_1.TIF]
