# Supplementary material for: The Involvement of the Chemokine RANTES in Regulating Luminal Acidification in Rat Epididymis
Source: Front Immunol. 2020 Sep 25;11:583274. doi: 10.3389/fimmu.2020.583274 (PMC7544837; doi:10.3389/fimmu.2020.583274)
Supplement: Supplementary file 2 [file Table_1.DOCX]

**Supplementary Methods**

**Table 1. Primers sequences used for RT-PCR**

| **Gene** | Forward primerReverseprimer |
| --- | --- |
| ***RANTES*** | 5΄-CTGCTGCTTTGCCTACCTCTC -3΄5΄- CTGCTGCTTTGCCTACCTCTC -3΄ |
| ***CCR1*** | 5΄-CCTACCCCACAACCACAGAATA -3΄5΄- CCACTCCAATGATAAACACGAA -3΄ |
| ***CCR3*** | 5΄-CTGTATTCCCTGGTGTTCATCGTCG -3΄5΄-CTGGACTCCCTACAACATTGTCCT -3΄ |
| ***CCR5*** | 5΄-CTGGACTCCCTACAACATTGTCCT -3΄5΄-TCCTGTTCTCCTGTGGACCG -3΄ |
| ***β****-actin* | 5΄-ACTGCCGCATCCTCTTCCTC -3΄5΄-ACTCCTGCTTGCTGATCCACA -3΄ |

**Table 2. Primers sequences used for qPCR**

| **Gene** | Forward primerReverseprimer |
| --- | --- |
| ***iNOS*** | 5΄- GTTTGACCAGAGGACCCAGA -3΄5΄- TCCTTTGTTACGGCTTCCAG-3΄ |
| ***V-ATPase*** | 5΄-GAGGAGACAAGATGGCGTTG -3΄5΄-ACGAGGCTGGGAGAGGTAGT -3΄ |
| ***RANTES*** | 5΄-CTGCTGCTTTGCCTACCTCTC -3΄5΄- CTTGAACCCACTTCTTCTCTGG-3΄ |
| ***CCR1*** | 5΄- CCTACCCCACAACCACAGAATA-3΄5΄-CCACTCCAATGATAAACACGAA-3΄ |
| ***CCR5*** | 5΄- ACTATTGGGATGACACACTGCT-3΄5΄- CAGGATTGACTTGCTGGAAAAT-3΄ |
| ***AGTR2*** | 5΄-TCCCTTCCTGTATTGTTTCGTT -3΄5΄-CATAGTCTCTCTCTTGCCTTGGA-3΄ |
| ***GAPDH*** | 5΄-GGCACAGTCAAGGCTGAGAAT -3΄5΄-ATGGTGGTGAAGACGCCAGTA -3΄ |
| ***β****-actin* | 5΄-ACTGCCGCATCCTCTTCCTC -3΄5΄-ACTCCTGCTTGCTGATCCACA -3΄ |
