# Supplementary material for: The Involvement of the Chemokine RANTES in Regulating Luminal Acidification in Rat Epididymis
Source: Front Immunol. 2020 Sep 25;11:583274. doi: 10.3389/fimmu.2020.583274 (PMC7544837; doi:10.3389/fimmu.2020.583274)
Supplement: Supplementary file 3 [file Table_2.DOCX]

SUPPLEMENTAL FIGURE | Cellular localization of iNOS in the adult rat epididymis. The cellular location of iNOS in caudal epididymis detected by double IF. The green signal of iNOS (CY3 labeled) coincide with the red signal of F4/80 (FITC labeled for macrophage).Bar=20 μm.

SUPPLEMENTAL TABLE | Primer sequences for RT-PCR and qPCR in this work.
